# Bariatric surgery increases the rate of major fracture: self‐controlled case series study in UK Clinical Practice Research Datalink

**DOI:** 10.1002/jbmr.4405

**Published:** 2021-07-29

**Authors:** Danielle E. Robinson, Ian Douglas, Garry D. Tan, Antonella Delmestri, Andrew Judge, Cyrus Cooper, M. Kassim Javaid, Victoria Y. Strauss, Daniel Prieto‐Alhambra

**Affiliations:** ^1^ Nuffield Department of Orthopaedics, Rheumatology and Musculoskeletal Sciences University of Oxford Oxford UK; ^2^ Department of Non‐Communicable Disease Epidemiology London School of Hygiene & Tropical London UK; ^3^ Oxford Centre for Diabetes, Endocrinology and Metabolism, Oxford University Hospitals National Health Service (NHS) Foundation Trust Oxford UK; ^4^ The National Institute for Health Research (NIHR) Oxford Biomedical Research Centre Oxford UK; ^5^ Medicine Musculoskeletal Research Unit, Translational Health Sciences, Bristol Medical School University of Bristol Bristol UK; ^6^ MRC Lifecourse Epidemiology Unit University of Southampton Southampton UK; ^7^ National Institute for Health Research (NIHR) Southampton Biomedical Research Centre University of Southampton and University Hospital Southampton UK; ^8^ National Institute for Health Research (NIHR) Oxford Biomedical Research Centre University of Oxford Oxford UK; ^9^ GREMPAL Research Group, Idiap Jordi Gol and CIBERFes Universitat Autonoma de Barcelona and Instituto de Salud Carlos III Barcelona Spain

**Keywords:** EPIDEMIOLOGY, GENERAL POPULATION STUDIES, FRACTURE RISK ASSESSMENT, NUTRITION, STATISTICAL METHODS

## Abstract

Conflicting results exist about the relationship between bariatric surgery and fracture risk. Also, prediction of who is at increased risk of fracture after bariatric surgery is not currently available. Hence, we used a combination of a self‐controlled case series (SCCS) study to establish the association between bariatric surgery and fracture, and develop a prediction model for postoperative fracture risk estimation using a cohort study. Patients from UK Primary care records from the Clinical Practice Research Datalink GOLD linked to Hospital Episode Statistics undergoing bariatric surgery with body mass index (BMI) ≥30 kg/m^2^ between 1997 and 2018 were included in the cohort. Those sustaining one or more fractures in the 5 years before or after surgery were included in the SCCS. Fractures were considered in three categories: (i) any except skull and digits (primary outcome); (ii) major (hip, vertebrae, wrist/forearm, and humerus); and (iii) peripheral (forearm and lower leg). Of 5487 participants, 252 (4.6%) experienced 272 fractures (of which 80 were major and 135 peripheral) and were included in the SCCS analyses. Major fracture risk increased after surgery, incidence rate ratios (IRRs) and 95% confidence intervals (CIs): 2.77 (95% CI, 1.34–5.75) and 3.78 (95% CI, 1.42–10.08) at ≤3 years and 3.1 to 5 years postsurgery when compared to 5 years prior to surgery, respectively. Any fracture risk was higher only in the 2.1 to 5 years following surgery (IRR 1.73; 95% CI, 1.08–2.77) when compared to 5 years prior to surgery. No excess risk of peripheral fracture after surgery was identified. A prediction tool for major fracture was developed using 5487 participants included in the cohort study. It was also internally validated (area under the receiver‐operating characteristic curve [AUC ROC] 0.70) with use of anxiolytics/sedatives/hypnotics and female as major predictors. Hence, major fractures are nearly threefold more likely after bariatric surgery. A simple prediction tool with five variables identifies high risk patients for major fracture. © 2021 The Authors. *Journal of Bone and Mineral Research* published by Wiley Periodicals LLC on behalf of American Society for Bone and Mineral Research (ASBMR).

## Introduction

Bariatric surgery has been proven to be a highly effective treatment for severe obesity with improvements in relevant clinical endpoints, such as a reduction in cardiovascular events and cardiovascular death.^(^ ^1^ ^)^ Bariatric surgery also leads to the remission of diabetes,^(^ ^2^ ^)^ remission of hypertension, and protection against obstructive sleep apnea. It has proved to be cost effective and is therefore recommended for the management of obesity in the UK (National Institute for Health and Care Excellence [NICE] Clinical Guideline 189 [CG189]^(^ ^3^ ^)^). However, there are concerns about the effects of bariatric surgery on bone health: recent analyses have suggested that patients who undergo bariatric surgery have an increased risk of postoperative fracture^(^ ^4^ ^)^; however, a systematic review and meta‐analysis of trials and observational studies has concluded no effect is currently shown, but it stated that more data is needed.^(^ ^5^ ^)^

Current evidence relies on cohort and case‐control studies, where differences in patient characteristics remain between those who undergo bariatric surgery and those who do not,^(^ ^6^ ^)^ suggesting this potential increased risk may be due to confounding. A more robust within‐person study design called self‐controlled case series (SCCS) provides an opportunity to investigate this association in a within‐person analysis, where such confounding is controlled for by design.^(^ ^7^ ^)^

In addition, the current NICE‐recommended fracture prediction tools^(^ ^8^ ^)^ are unlikely to be valid for the identification of patients undergoing bariatric surgery who might need further monitoring/testing (e.g., bone mineral density scans or serum measurement/s of vitamin D levels). In fact, both the Fracture Risk Assessment Tool (FRAX)^(^ ^9^ ^)^ and QFracture^(^ ^10^ ^)^ tools assume a protective effect of obesity on fracture risk, demonstrating the need for a bespoke fracture prediction tool for the identification of patients undergoing bariatric surgery at need of further evaluation and/or treatment of their bone health postoperatively.

We aimed to first study the effect of bariatric surgery on postoperative fracture risk in National Health Service (NHS) patients with obesity using SCCS. Second, we set out to identify key determinants of postoperative fracture risk, and to combine them to derive a prediction tool for the identification of patients at high risk of such fractures.

## Materials and Methods

### Study design and data sources

Two retrospective studies, one SCCS and a cohort, were undertaken using primary care records from the Clinical Practice Research Datalink (CPRD) GOLD with linkage to hospital inpatient records, hospital episode statistics (HES), in England between April 1, 1997 and May 1, 2018. These datasets contain anonymized primary and secondary care records. CPRD‐HES contains records from 401 practices, covering approximately 58% of CPRD‐registered practices, whereas CPRD without linkage contains information on a further 263 practices. Both have previously been shown to be representative of patients.

Scientific approval was given for this study, CPRD protocol number 17_258.

### Eligibility criteria

Patients were eligible for inclusion if they underwent bariatric surgery at age ≥18 years, recorded in either their primary (READ code) or hospital (OPCS‐4 code) record. Code lists of the bariatric surgeries are provided in GitHub (https://github.com/daniellerobinson10/bariatric_codelist). Patients were required to have a preoperative body mass index (BMI) of ≥30 kg/m^2^, and no history of gastric cancer prior to the surgery.

### Exposure: bariatric surgery

Bariatric surgery records were identified either in the primary care using a previously published list of READ codes^(^ ^11^ ^)^ or hospital records using HES OPCS‐4 codes. To avoid duplication of surgery codes, codes within 1 year of each other were considered to be the same surgery.

### Outcome of interest

Fractures were identified from primary care records (READ codes) in the 5 years before (only for SCCS) or after surgery, and classified under three categories: any fracture (primary, any location except skull or digits), major fracture (hip, spine, wrist/forearm, or proximal humerus), and peripheral fracture (forearm or lower leg) using an updated, for completeness, version of a previously validated list of READ codes.^(^ ^12^ ^)^ Fractures in the same skeletal site occurring at least 3 months apart and fractures at different locations were considered separate fracture occurrences.

### Candidate preoperative predictors of postoperative fracture (cohort study only)

Candidate predictors were identified within a consensus meeting between two clinicians (a general practitioner with expertise in fracture prevention [Daniel Prieto‐Alhambra], a bone specialist [M. Kassim Javaid]). These predictors included known fracture risk factors; for example, age and previous systematic steroid use, type 2 diabetes, use of other drugs to prevent fractures, and socioeconomic deprivation (index of multiple deprivation), all derived from CPRD. Supplement S2 includes descriptions of the candidate predictor.

### Statistical analysis

#### Self‐controlled case series

The SCCS was undertaken on patients who experience fracture for each of the three fracture categories individually, descriptive statistics were produced for both the full cohort of bariatric surgery patients and patients who experienced each individual fracture type. Numbers and percentages of categorical variables are presented alongside mean and standard deviation of normally distributed continuous variables and median and interquartile range of skewed continuous variables. Incidence rates of 5 years before (reference) and after surgery were compared and incidence rate ratios (IRRs), calculated with a conditional Poisson model. We controlled for increases in age (an ordinal variable), and time‐varying bisphosphonate use (ever used). Three patterns of postoperative exposed time windows were analyzed: (i) 0–5 years, (ii) 0–3 and 3.01–5 years, and (iii) 0–2 and 2.01–5 years (a post hoc analysis). The post hoc analysis was added because patients are released from endocrinology care after 2 years in the UK. Patients could have multiple surgeries, but gaps of at least 1 year were required between included surgeries. Patients with multiple surgeries <5 years apart were followed until 5 years after the last surgery. Three SCCS assumptions were tested^(^ ^13^, ^14^ ^)^ using histograms and sensitivity analyses explained in Supplement S1. Other sensitivity analysis included tests for interaction between bariatric surgery and sex, type 2 diabetes diagnosis, surgery type, and weight‐loss quartile in the first year postsurgery with a missing category if there was no weight information postsurgery. Stratified results are reported for interactions where *p* < 0.1.

#### Cohort

For this analysis only the first postoperative fracture per patient in each of the three categories was included. For each candidate predictor, we reported its unadjusted association with outcomes using univariate logistic regression. Stepwise logistic regressions with backward elimination were then applied to selected risk factors of postoperative fracture in the final multivariate model. Age and weight loss in the prior year was included as a continuous variable, whereas BMI was log‐transformed so it best explained the association between BMI and risk of fracture. Missing data for potential predictors of smoking and drinking, ethnicity, and marital status were imputed using multiple imputation by chained equations with 20 imputations. Predictors were selected with an exit *p* value of 0.157^(^ ^15^ ^)^ and retained if they met this criterion in ≥80% of the 20 imputed models. Rubins rules were only applied in the model with predictors that fulfilled the selection criteria above.

Bootstrap validation with replacement (500 iterations) was applied for internal validation. Predictive performance was assessed using the area under the receiver‐operating characteristic (AUC ROC) curve.^(^ ^16^ ^)^ To account for overfitting, we reported optimism‐adjusted (difference in the test and development performance) AUC. The accuracy of the model performance was tested using calibration plots of expected versus observed risk, and derived calibration slopes (where 1 is ideal).

All statistical analyses were undertaken in Stata version 15.1 (StataCorp, LLC, College Station, TX, USA). The reporting guidelines of the Strengthening the Reporting of Observational Studies in Epidemiology (STROBE) statement were followed.

### Sample size consideration

The sample size for objective 1 was calculated a priori. According to the method proposed by Musonda et al.^(^ ^17^ ^)^ and implemented in the sampsi sccs command in Stata,^(^ ^18^ ^)^ 68 patients with any fracture would be needed to detect as significant an IRR of ≥2 in a two‐sided SCCS analysis with alpha of 0.05, 80% power, and a 5‐year (postsurgery) exposure period.

### Role of the funding source

No funder was directly involved in any aspect of this work. The views expressed are those of the authors and not necessarily those of the NHS, the National Institute for Health Research (NIHR), or the Department of Health and Social Care.

## Results

**FIGURE 1:**
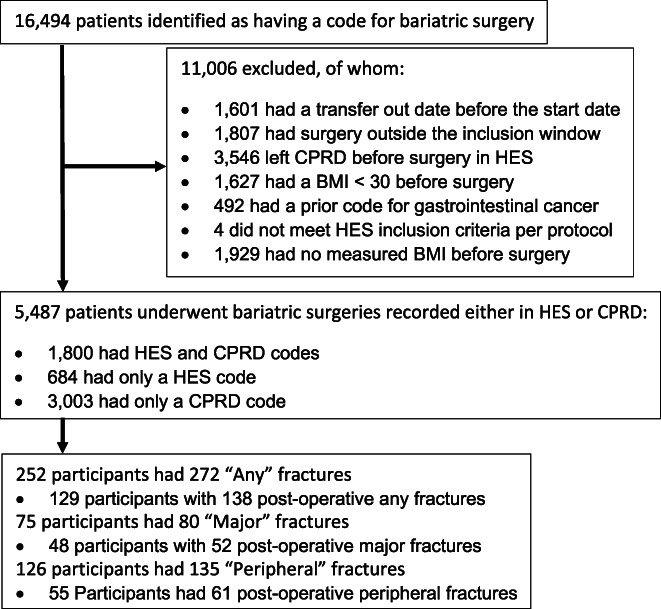
Flow diagram of exclusions from the cohort and numbers included for each self controlled case series analysis. Abbreviations: BMI, body mass index; CPRD, Clinical Practice Research Datalink; HES, hospital episode statistics.

Of 16,493 patients identified with a code for bariatric surgery, 5487 patients were deemed suitable for study inclusion. Figure 1 shows the exclusions. Note: 3546 patients were excluded because their surgery (identified in HES) occurred after they had left their CPRD practice or their practice had stopped providing data to CPRD hence no information is available about the patients covariates or outcome. Participants undergoing bariatric surgery were on average 40 years old (age range 18–85 years). Most were female (77.8%), with a median (interquartile range) BMI of 43.9 (38.7–49.7) kg/m^2^ before surgery (Table 1). Nearly 30% had type 2 diabetes. The most common surgeries were “partition surgeries” (42.4%) and “bypass surgeries” (35.0%). Participants who had a major or any included fracture were older, more likely to smoke, be female, and have used anti‐depressants in the past year.

**TABLE 1:** Baseline (date of surgery) characteristics of the whole cohort, and those with any, major, and peripheral fractures

Fractures included	All	Any fractures (All fractures except skull and digits)	Major fractures (Hip, spine, forearm, and shoulder)	Peripheral fractures (Forearm and lower leg)
Patients (*n*)	5487	252	75	126
Age (years), mean ± SD	40.7 ± 10.7	42.4 ± 10.8	43.0 ± 10.0	40.9 ± 10.1
Gender (female), *n* (%)	4269 (77.8)	193 (76.6)	63 (84.0)	101 (80.2)
BMI (m/kg^2^), median (IQR)	43.9 (38.7, 49.7)	44.0 (38.9, 49.6)	45.3 (40.4, 49.7)	44.6 (40.2, 49.6)
IMD quintiles, *n* (%)				
1 (least deprived)	716 (13.0)	38 (15.1)	7 (9.3)	19 (15.1)
2	766 (14.0)	34 (13.5)	10 (13.3)	16 (12.7)
3	738 (13.4)	24 (9.5)	7 (9.3)	15 (11.9)
4	889 (16.2)	33 (13.1)	8 (10.7)	15 (11.9)
5 (most deprived)	676 (12.3)	29 (11.5)	9 (12.0)	15 (11.9)
Missing	1702 (31.0)	94 (37.3)	34 (45.3)	14 (11.1)
Ethnicity, *n* (%)				
White	4181 (76.2)	197 (78.2)	54 (72.0)	94 (74.6)
Not white	288 (5.2)	55 (21.8)	21 (28.0)	31 (24.6)
Missing	1018 (18.6)	0	0	<5
Smoking status, *n* (%)				
Yes	734 (13.4)	46 (18.3)	16 (21.3)	24 (19.0)
No	2686 (49.0)	114 (45.2)	33 (44.0)	56 (44.4)
Ex‐smoker	1902 (34.7)	89 (35.3)	26 (34.7)	45 (35.7)
Missing	165 (3.0)	<5	0	<5
Drinking status, *n* (%)				
Yes	3037 (55.3)	148 (58.7)	49 (65.3)	72 (57.1)
No	932 (17.0)	39 (15.5)	11 (14.7)	21 (16.7)
Ex‐drinker	408 (7.4)	23 (9.1)	5 (6.7)	11 (8.7)
Missing	1110 (20.2)	42 (16.7)	10 (13.3)	22 (17.5)
First surgery type, *n* (%)				
Gastrectomy	1067 (19.4)	42 (16.7)	10 (13.3)	18 (14.3)
Partition	2324 (42.4)	106 (42.1)	31 (41.3)	56 (44.4)
Balloon	177 (3.2)	<5	0	<5
Bypass	1919 (35.0)	102 (40.5)	34 (45.3)	51 (40.5)
History of fracture before surgery, *n* (%)	258 (4.7)	126 (50.0)	28 (37.3)	69 (54.8)
Rheumatoid arthritis, *n* (%)	68 (1.2)	<5	<5	<5
Type 2 diabetes, *n* (%)	1621 (29.5)	85 (33.7)	23 (30.7)	46 (36.5)
Type 1 diabetes, *n* (%)	43 (0.8)	<5	<5	<5
Osteogenesis imperfecta, *n* (%)	0 (0.0)	0 (0.0)	0 (0.0)	0 (0.0)
Menopause, *n* (%)	478 (8.7)	33 (13.1)	10 (13.3)	17 (13.5)
Steroids in past year, *n* (%)	656 (12.0)	40 (15.9)	14 (18.7)	20 (15.9)
Antiepileptics in past year, *n* (%)	443 (8.1)	14 (5.6)	3 (4.0)	7 (5.6)
Antidepressants in past year, *n* (%)	2037 (37.1)	102 (40.5)	36 (48.0)	59 (46.8)
Anxiolytics/sedatives/hypnotics in past year, *n* (%)	524 (9.5)	31 (12.3)	13 (17.3)	14 (11.1)
Calcium and vitamin D in past year, *n* (%)	507 (9.2)	28 (11.1)	11 (14.7)	12 (9.5)
Bisphosphonates in past year, *n* (%)	43 (0.8)	<5	<5	0

*Notes*: Where <5 events occurred the value <5 is shown. This is a guideline required for the reporting of numbers specified by the holders of CPRD data.Abbreviations: BMI, body mass index; CPRD, Clinical Practice Research Datalink; IMD, index of multiple deprivation; IQR, interquartile range; SD, standard deviation.

Of 5487 patients undergoing bariatric surgeries, 1800 had bariatric codes both in primary and secondary care of a total 3694 surgeries where patients had HES linkage. The inclusion of HES codes identified 452 more patients with bariatric surgery than the CPRD list alone.

### Self‐controlled case series

Of the 5487 participants, 252 had 272 any fractures, 75 had 80 major fractures, and 126 had 135 peripheral fractures. To be eligible for the SCCS patients had to have a fracture of interest prior to surgery or postsurgery in the analysis; hence, the sample sizes were 252 patients for the any fracture analysis, 75 for the major fracture analysis, and 126 for the peripheral fracture analysis. Table 2 shows the results of the SCCS analysis for the three fracture groupings and three prespecified time windows.

**TABLE 2:** Incidence rate ratios and 95% CIs of the self‐controlled case series analysis

		Incidence rate ratios for each postsurgery exposed time versus 5‐year prior surgery unexposed time 95% CIs					
Fracture location	Average follow‐up postsurgery median (IQR)	0–5 years unadjusted	0–5 years adjusted^a^	0–3 years adjusted^a^	3.01–5 years adjusted^a^	0–2 years adjusted^a^	2.01–5 years adjusted^a^
Any	4.6 (2.4, 5.0)	1.57 (1.25, 1.98)	1.17 (0.86, 1.60)	1.20 (0.83, 1.72)	1.33 (0.79, 2.22)	1.11 (0.75, 1.62)	1.73 (1.08, 2.77)
Major	4.9 (2.4, 5.0)	3.12 (1.87, 5.21)	2.70 (1.31, 5.57)	2.77 (1.34, 5.75)	3.78 (1.42, 10.1)	2.49 (1.17, 5.30)	4.98 (1.94, 12.8)
Peripheral	4.6 (2.3, 5.0)	1.49 (1.08, 2.04)	0.92 (0.60, 1.42)	0.85 (0.50, 1.46)	1.06 (0.53, 2.20)	0.75 (0.43, 1.33)	1.18 (0.60, 2.30)

Abbreviations: CI, confidence interval; IQR, interquartile range.

^a^ Adjusted for age (in 5‐year bands) and bisphosphonate use.

The rate of major fracture was nearly threefold increased, with an IRR (95% confidence interval [CI]) of 2.70 (95% CI, 1.31–5.57) in the first 5 years after bariatric surgery, compared to the 5 years before surgery. The relative incidence of major fractures was highest in the 2 to 5 years postsurgery (IRR 4.98; 95% CI, 1.94–12.78). The relative incidence of any fractures also increased for the 2 to 5 years postsurgery (1.73; 95% CI, 1.08–2.77). The relative incidence of peripheral fractures did not increase in the 5 years after bariatric surgery, compared to before surgery.

When interactions were tested, only that in the major fracture model, between bariatric surgery and sex was statistically significant (*p* = 0.024). The IRR (95% CI) of major fractures was 3.31 (95% CI, 1.56–7.51) in women and 0.71 (95% CI, 0.11–4.68) in men. This analysis was limited by the low number of fractures (12) in men. No interaction was identified with type 2 diabetes diagnosis, surgery type, and weight‐loss quartile.

### Fracture risk prediction modeling

In the 5‐year postsurgery period, 129 patients had at least one of any fracture giving a cumulative incidence (95% CI) of 23.5 of 1000 patients (95% CI, 19.6–27.9). Similarly, the number and cumulative incidence (95% CI) were 48 and 8.7 (95% CI, 6.5–11.6) for major fractures and 55 and 10.0 (95% CI, 7.6–13.0) for peripheral fractures, respectively.

**FIGURE 2:**
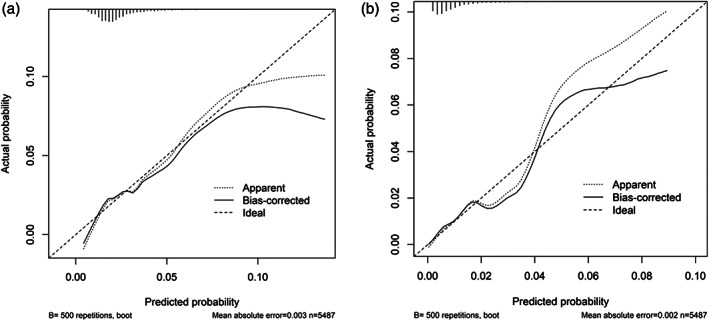
Calibration plots for the prediction of (*A*) any and (*B*) major fractures with expected and observed numbers and (percentages) for each quintile. Quintile 3 of any fractures were outside the range of expected values

Results of the univariate logistic regression are shown in Supplement S3. In the multivariate model, of all the potential predictors considered, five were retained after backward elimination for the prediction of postsurgery major fracture: age, females, region/country of residence, use of antiepileptics or anxiolytics/sedatives/hypnotics, and use of antidepressants in the past year (Table 3). Female sex (adjusted odds ratio [OR] 3.32; 95% CI, 1.18–9.36]) was the strongest predictor for major fracture, followed by use of anxiolytics/sedatives/hypnotics (2.56; 95% CI, 1.29–5.05) and age (1.23; 95% CI, 1.09–1.40) per 5‐year increase. The final model had an optimism‐adjusted AUC of 0.70 (95% CI, 0.63–0.76; Supplement S4) and the calibration plot and calibration slope of 0.83 indicate good calibration (Figure 2*B*).

**TABLE 3:** Multivariate logistic regression associations with fracture and β coefficients of the predictors included in the final model

Parameter	Any OR (95% CI)	β coefficient	Major OR (95% CI)	β coefficient	Peripheral OR (95% CI)
Age per 5 years	1.16 (1.07, 1.25)	0.14 (0.07, 0.22)	1.23 (1.09, 1.40)	0.21 (0.08, 0.34)	1.12 (0.99, 1.26)
Gender (female)	‐	‐	3.32 (1.18, 9.36)	1.20 (0.16, 2.24)	‐
Region					
South	Ref	Ref	Ref	Ref	‐
London	1.00 (0.58, 1.74)	0.00 (−0.55, 0.55)	0.36 (0.10, 1.22)	−1.03 (−2.27, 0.20)	
East	0.46 (0.18, 1.18)	−0.77 (−1.71, 0.16)	0.90 (0.30, 2.71)	−0.10 (−1.20, 1.00)	
West	0.88 (0.54, 1.43)	−0.12 (−0.61, 0.36)	0.80 (0.37, 1.72)	−0.22 (−0.99, 0.54)	
Scotland	2.36 (1.39, 4.03)	0.86 (0.33, 1.39)	2.22 (0.98, 5.04)	0.80 (−0.02, 1.62)	
Wales	1.27 (0.66, 2.44)	0.24 (−0.42, 0.89)	1.00 (0.33, 2.99)	0.00 (−1.10, 1.62)	
Northern Ireland	0.71 (0.10, 5.28)	−0.34 (−2.35, 1.66)	No events	No events	
Smoking status					
Yes	Ref	Ref	‐	‐	‐
No	0.57 (0.35, 0.92)	−0.56 (−1.04, −0.08)			
Ex‐smoker	0.59 (0.36, 0.97)	−0.53 (−1.03, −0.03)			
History of fracture (yes)	2.02 (1.09, 3.74)	0.70 (0.09, 1.32)	‐	‐	‐
Antiepileptics prior year (yes)	0.54 (0.25, 1.18)	−0.62 (−1.4, 0.16)	‐	‐	‐
Antidepressants prior year (yes)			1.66 (0.92, 3.00)	0.51 (−0.08, 1.10)	1.67 (0.98, 2.84)
Anxiolytics/sedatives/hypnotics prior year (yes)	1.74 (1.06, 2.87)	0.56 (0.06, 1.06)	2.56 (1.29, 5.05)	0.94 (0.26, 1.62)	‐

*Notes*: Both OR and β coefficients are included in line with TRIPOD guidelines. ORs report no difference when the value 1 is included in the 95% CI whereas β coefficients report no difference when the value 0 is included in the 95% CI. When comparing the OR and β coefficients, the beta for the association between age and any fracture is the log OR of 0.14. The OR is the exponentiated coefficient of 0.14; that is, exp(0.14) giving an OR of 1.16 for the same association between age and any fracture showing that for each 5‐year increase the risk of any fracture increase by 16%.

Abbreviations: CI, confidence interval; OR, odds ratio; TRIPOD, transparent reporting of a multivariable prediction model for individual prognosis or diagnosis.

All risk factors included in the postoperative major fracture prediction model except for females and use of antidepressants were retained in the prediction model for any postoperative fracture and smoking status and use of anti‐epileptic medication were included. Model discrimination was poor in the internal bootstrap validation with an optimism‐adjusted AUC of 0.61 (95% CI, 0.57–0.65; Supplement S4). Low prediction accuracy and a potential of overfitting were observed in Figure 2*A*, particularly when there was >10% predicted risk, and by the calibration slope of 0.82 (95% CI, 0.70–1.30).

Finally, only age and use of antidepressants presurgery were associated with peripheral fracture risk (Table 3). The derived model had poor discrimination with an AUC of 0.59 (95% CI, 0.51–0.67) (data not shown).

## Discussion

Our findings confirm an excess risk of major and any fracture, but not peripheral fracture in the 5 years following bariatric surgery for patients with obesity. Using an SCCS analysis, we have identified a nearly threefold excess risk of major fracture within the 5 years after bariatric surgery, compared with the 5 years presurgery. When these time windows were split into the first 2 years postsurgery and final 3 years postsurgery, the later time window had a nearly fivefold increased incidence compared to the baseline (presurgery) risk. The incidence of any fractures was only significantly increased in the 2‐year to 5‐year postoperative window, possibly driven by the substantial increase in risk of major fractures.

In addition, we have identified key determinants of postsurgery fracture risk. A prediction tool based on the combination of five variables (age, female sex, use of sedatives/hypnotics, use of antidepressants, and region of residence) had reasonable performance for the identification of patients at high risk of major fracture, with clinically acceptable discrimination and good calibration. Most of the identified predictors were similar to those in general risk prediction models such as FRAX^(^ ^9^ ^)^ or QFracture,^(^ ^10^ ^)^ whereas others confer additional risk, including the recent use of sedatives. Of interest, some predictors, such as history of fracture, were not identified in the prediction of major fracture. However, this is likely due to the lack of statistical power. Four of 48 patients who had a major fracture had a history of fracture resulting in a univariate OR of 1.86 with a wide confidence interval of 0.66 to 5.20.

Little is known about the etiopathogenesis of bone fragility in patients with obesity. A number of studies have suggested that obesity is protective against hip fractures,^(^ ^19^ ^)^ but might lead to an increased risk of peripheral fractures (wrist, shoulder, or ankle),^(^ ^20^, ^21^ ^)^ postfracture complications, and even mortality.^(^ ^22^ ^)^

The mechanisms behind the identified increased incidence and potential predictors of major fractures are not known.^(^ ^23^ ^)^ After bariatric surgery, patients experience nutritional deficits, including deficits in the absorption of calcium and vitamin D, key nutrients for bone health. Furthermore, weight loss after bariatric surgery may decrease the expression of sclerostin due to reduced mechanical loading resulting in upregulation of the Wnt/beta‐catenin pathway increasing bone resorption. Finally, the reduction in weight can lead to an increase in physical exercise for the patient increasing the risk of falls and fractures. In the UK, it is advised that all patients receive a minimum of 800 to 1200 mg calcium and 20 μg (800 IU) vitamin D per day^(^ ^24^ ^)^; however, only 29.3% of participants had a calcium and vitamin D prescription at 6 months postsurgery and 21.5% at 5 years postsurgery. This supports the above hypothesis that malabsorption of calcium and vitamin D postsurgery may be increasing the risk of fracture through reduced prescription of supplements. The data in CPRD‐HES does not allow for the testing of the other two mechanisms of increased fracture risk.

The finding that sedative and antidepressant use predicted a higher risk of fractures is important as this is a modifiable risk factor and there was an extremely high prevalence of use of these drugs in this analysis. It would be reasonable to recommend a review of risks and benefits of sedatives and of antidepressants in patients who are planning bariatric surgery. The finding that fracture risk postsurgery was higher in Scotland versus other regions is in line with previous data on the epidemiology of fragility fractures in the UK.^(^ ^25^ ^)^ This may reflect differences in the healthcare systems in different parts of the UK and needs further investigation.

The SCCS analysis supports previous cohort studies comparing the risk of fracture in patients undergoing bariatric surgery compared to those who do not. These studies have shown that fracture risk increases after surgery and with time.^(^ ^26^, ^27^ ^)^ The identification of an excess incidence of major fracture using both cohort studies and a within person study designs in cohorts throughout the world suggest this is a real effect and further work should be undertaken to identify the mechanism behind the increased fracture incidence. The lack of result in the analysis in peripheral fracture may be because it has previously been shown that risk of lower leg fracture decreases after bariatric surgery whereas the risk of forearm fracture increases.^(^ ^6^ ^)^ Hence the risk of fracture at one location of surgery may be cancelling out the other.

The risk prediction model needs further validation in an external dataset, due to the low number of fractures in our cohort allowing for internal validation, before it can be applied to a clinical setting.

The findings of this work suggest that patients undergoing bariatric surgery should be assessed for their risk of fracture. In particular fracture of the hip, vertebrae, proximal humerus, or radius/ulna. Vitamin D and calcium levels should be assessed in patients who have undergone bariatric surgery, in particular once discharged to primary care, to ensure any malabsorption due to the surgery is identified and preventative treatment of fractures can be considered.

This study has limitations. Missing marital status and ethnicity were imputed with an assumption of missing at random, though evidence showed otherwise.^(^ ^28^, ^29^ ^)^ Although we used BMI >30 kg/m^2^ as an inclusion criterion, NICE recommends bariatric surgery for people either with BMI >35 kg/m^2^ and obesity related comorbidities or with a BMI >40 kg/m^2^ without. We used this relaxed criterion to account for privately funded bariatric surgery, which may be identified in CPRD only. We did not impute the index of multiple deprivation (IMD) because predictors of missing IMD depended mainly on socioeconomic data that were not available for routinely collected data. IMD was not included as a separate predictor because it was highly correlated with region. Because age was only adjusted in the SCCS using an ordinal variable of 5‐year age bands due to a lack of events, we may not have fully adjusted for age in the model. There was also a risk of overfitting in the prediction modeling due to the small number of events in the analysis of major and peripheral fracture; however, the internal validation did not significantly affect the AUC for the major fracture analysis. The CPRD does not capture all predictors of fracture risk, such as risk‐seeking behavior, baseline vitamin D level, and baseline bone mineral density. Also, there might have been some risk factors that could predict postsurgical fracture which we have missed out in our prediction model; however, due to the low number of postsurgical fractures, it would be unlikely to make significant difference in our derived prediction model. Finally, the lack of events prevented the analysis of fracture incidence in different types of bariatric surgery. In the major fracture interaction analysis, only five postoperative major fractures occurred in a patient who had a gastrectomy, 25 in a patient who had a partition surgery, and 24 in a patient who had a bypass. Hence, no conclusion can be drawn about the association between surgery type and fracture risk, a question of clinical importance.

Our study has demonstrated an excess risk of fractures following bariatric surgery, and identified key determinants of postoperative fracture risk. The combination of five clinical variables successfully identifies subjects at increased risk of postoperative fractures. This tool warrants further external validation. Once done, it could be used to target patients undergoing bariatric surgery who might require additional investigations (e.g., bone densitometry scans) and/or monitoring. More research is needed to determine the risk‐benefit of calcium and vitamin D supplementation following bariatric surgery.

## Disclosures

All authors have completed the ICMJE uniform disclosure form at www.icmje.org/coi_disclosure.pdf and declare: Daniel Prieto‐Alhambra reports grants and other from AMGEN; grants, nonfinancial support, and other from UCB Biopharma; grants from Les Laboratoires Servier, outside the submitted work; he also reports that Janssen, on behalf of the IMI‐funded EHDEN and EMIF consortiums, and Synapse Management Partners have supported training programs that are organized by his department and are open to external participants. Andrew Judge reports grants from NIHR HTA, during the conduct of the study; personal fees from Freshfields Bruckhaus Derringer, personal fees from Anthera Pharmaceuticals LTD, outside the submitted work. Cyrus Cooper reports personal fees from Amgen, personal fees from Danone, personal fees from Eli Lilly, personal fees from GSK, personal fees from Kyowa Kirin, personal fees from Medtronic, personal fees from Merck, personal fees from Nestle, personal fees from Novartis, personal fees from Pfizer, personal fees from Roche, personal fees from Servier, personal fees from Shire, personal fees from Takeda, personal fees from UCB, outside the submitted work. Antonella Delmestri, Danielle E. Robinson, Victoria Y. Strauss, M. Kassim Javaid, Ian Douglas, and Garry D. Tan have nothing to disclose. The corresponding author attests that all listed authors meet authorship criteria and that no others meeting the criteria have been omitted.

## Author Contributions

Danielle E Robinson: Formal Analysis, Investigation, Methodology, Software, Validation, Visualization, Writing ‐ Original Draft Preparation and Writing ‐ Review & Editing. Ian Douglas: Conceptualization, Funding Acquisition, Methodology, Supervision, Writing ‐ Review & Editing. Garry D Tan: Conceptualization, Funding Acquisition, Writing ‐ Reviewing & Editing. Antonella Delmestri: Conceptualization, Data Curation, Funding Acquisition, Software, Writing ‐ Reviewing & Editing. Andrew Judge: Conceptualization, Funding Acquisition, Methodology, Supervision, Writing ‐ Review & Editing. Cyrus Cooper: Conceptualization, Funding Acquisition, Writing ‐ Reviewing & Editing. M Kassim Javaid: Conceptualization, Funding Acquisition, Writing ‐ Reviewing & Editing. Victoria Y Strauss: Formal Analysis, Investigation, Methodology, Software, Supervision, Validation, Visualization, Writing ‐ Original Draft Preparation and Writing ‐ Review & Editing. Daniel Prieto‐Alhambra: Conceptualization, Funding Acquisition, Investigation, Supervision, Writing ‐ Original Draft Preparation and Writing ‐ Review & Editing.

## Additional Statements

The Corresponding Author has the right to grant on behalf of all authors and does grant on behalf of all authors, a worldwide license to the Publishers and its licensees in perpetuity, in all forms, formats and media (whether known now or created in the future), (i) to publish, reproduce, distribute, display and store the Contribution; (ii) to translate the Contribution into other languages, create adaptations, reprints, include within collections and create summaries, extracts, and/or abstracts of the Contribution; (iii) to create any other derivative work(s) based on the Contribution; (iv) to exploit all subsidiary rights in the Contribution; (v) the inclusion of electronic links from the Contribution to third party material wherever it may be located; and (vi) to license any third party to do any or all of the above.

### Peer Review

The peer review history for this article is available at https://publons.com/publon/10.1002/jbmr.4405.

## Supporting information


**Appendix S1**. Supporting Information

## Data Availability

Data was obtained from CPRD under Oxford University CPRD license and ISAC approval. Direct data sharing is not allowed, however, data access can be obtained from CPRD conditionally by an ISAC approval.
