# Supplementary material for: Bariatric surgery increases the rate of major fracture: self‐controlled case series study in UK Clinical Practice Research Datalink
Source: J Bone Miner Res. 2021 Jul 29;36(11):2153–61. doi: 10.1002/jbmr.4405 (PMC9290510; doi:10.1002/jbmr.4405)

**Supplement 1: SCCS assumption**

There are 4 assumptions of the SCCS: 1) the event does not temporally decreases/increases the probability of exposure; 2) event does not increase the probability of death; 3) the recurrence of an event is independent; 4) the event is not one whereby no exposure can happen after the event^1^. The first assumption was tested using a histogram in time between surgery and fracture and a comparison of IRR between the original model (with the 5-year post-operative as a time-at-risk window) and a model with a washout period of 8-week pre-exposed. The second assumption was tested by restricting to ‘alive’ cohort. The third assumption was ensured to hold by restricting the first fracture. The final assumption was not tested as the end of observation period is not related to event in our study.

Results:

A uniform distribution (supplementary Figure 1) in the time between surgery and fracture suggested no association between surgery date and fracture. In addition, the 8-week pre-exposed time appeared to have non-significant risk of developing any fracture in 5-year following surgery, with an IRR of 0.76 (95% CI: 0.38, 1.52) and the relative risk of any fracture in the 5 years after surgery when the 8 week pre-exposed time is removed from baseline was 1.14 (0.78, 1.50); similar to that found in the original model, 1.17 (0.86, 1.60). These indicated that occurrence of an event of any fracture did not affect subsequent exposure.

6% of patients undergoing bariatric surgeries died in the 5-year following bariatric surgeries. The “alive” cohort had similar IRR of 1.20 (0.83, 1.74) to the original IRR, 1.17 (0.86, 1.60) for developing any fracture in 5 years after surgeries, implying no association between fracture and early departure from the study.

Few people had multiple fractures. The ‘first fracture’ cohort yielded an IRR of 1.10 (0.76, 1.61) when compared to that in the original model, suggesting that events were independently recurrent. This is likely due to the small number of patients fracturing and the relatively young age of patients, mean age 40.7.

Supplementary figure 1: histogram of difference in years between surgery and any fracture.

**Reference:**

1. Petersen I, Douglas I, Whitaker H. Self controlled case series methods: an alternative to standard epidemiological study designs. *BMJ* 2016;354:i4515. doi: 10.1136/bmj.i4515

**Supplement 2:** Candidate predictors for the risk prediction models and how each candidate predictor is defined

| Covariate name | Combined variables | Contiuous measure | Categories | Combined categories |
| --- | --- | --- | --- | --- |
| Age | Age | Per 5 years | - | - |
| Gender | Gender | - | Male  Female | - |
| BMI | BMI | Log(BMI) per unit increase | - | - |
| Ethnicity | Ethnicity | - | White Not white | White  Black, Asian, Chinese, Mixed, Other |
| Region | Region | - | South  London  East  West  Scotland  Wales  N. Ireland | South Central, South East coast, South West  London  East Midlands, East of England, North East, Yorkshire & the Humber  North West, West Midlands  Scotland  Wales  Northern Ireland |
| Smoking status | Smoking status | - | Yes  No  Ex | - |
| Drinking status | Drinking status | - | Yes  No  Ex | - |
| Marital status | Marital status | - | Partnered  Single  Unknown | Married, Co-habiting, Remarried, Engaged, Civil Partnership  Single, Separated, Divorced, Widowed  Data not Entered, Missing |
| History of fracture | History of fracture |  | Yes  No | NA |
| Arthritis | Inflammatory arthritis  Rheumatoid arthritis | - | Yes  No | NA |
| Menopausal status | Premature menopause  Menopause | - | Yes  No | NA |
| Other conditions | Hyperthroidism  Hypogonadism  Malabsorption  Chronic liver disease | - | Yes  No | NA |
| Bone medications | Calcium & vitamin D  Bisphosphonates  Non bisphosphonates | - | Yes (past year)  No | NA |
| Steroid use | Steroid use | - | Yes (past year)  No | NA |
| Antiepileptic use | Antiepileptic use | - | Yes (past year)  No | NA |
| Antidepressant use | Antidepressant use |  | Yes (past year)  No | NA |
| Anxioltics/sedatives/ hypnotics use | Anxioltics/sedatives/ hypnotics use |  | Yes (past year)  No | NA |
| Diabetes | Type 1 diabetes  Type 2 diabetes  Insulin  Antidiabetics |  | Yes  No | NA |
| Kg weight loss in year prior to surery | Kg weight loss in year prior to surery | Per kg decrease | - | - |

**Supplement 3:** Univariate logistic regression association with fracture.

| Covariate | Category | Univariate prediction | | |
| --- | --- | --- | --- | --- |
|  |  | Any  (OR (95% CI)) | Major  (OR (95% CI)) | Peripheral  (OR (95% CI)) |
| Age | per 5 years | **1.15 (1.07, 1.24)** | **1.20 (1.06, 1.36)** | 1.11 (0.98, 1.25) |
| Gender | Female | 1.13 (0.73, 1.75) | **3.16 (1.13, 8.81)** | 1.29 (0.65, 2.56) |
| Ethnicity | White Not white | Ref  0.56 (0.19, 1.66) | Ref  0.51 (0.07, 3.68) | Ref  0.49 (0.07, 3.22) |
| Log(BMI) | NA | 0.83 (0.46, 1.65) | 1.25 (0.41, 3.73) | 1.38 (0.49, 3.83) |
| Region | South  London  East  West  Scotland  Wales  N. Ireland | Ref  1.00 (0.58, 1.73)  0.45 (0.18, 1.15)  0.87 (0.54, 1.41)  **2.35 (1.39, 3.99)**  1.25 (0.65, 2.40)  0.67 (0.09, 4.95) | Ref  0.38 (0.10, 1.29)  0.88 (0.29, 2.62)  0.80 (0.37, 1.70)  **2.28 (1.01, 5.15)**  1.00 (0.33, 2.98)  No events | Ref  0.64 (0.26, 1.61)  0.19 (0.02, 1.38)  0.80 (0.40, 1.61)  1.50 (0.63, 3.56)  1.49 (0.63, 3.55)  1.39 (0.18, 10.5) |
| Smoking status | Yes  No  Ex | Ref  **0.59 (0.37, 0.95**)  0.65 (0.40, 1.06) | Ref  0.78 (0.35, 1.75)  0.82 (0.35, 1.90) | Ref  0.65 (0.31, 1.37)  0.81 (0.38, 1.72) |
| Drinking status | Yes  No  Ex | Ref  0.78 (0.46, 1.30)  1.21 (0.66, 2.20) | Ref  0.78 (0.35, 1.77)  1.24 (0.49, 3.16) | Ref  0.91 (0.44, 1.89)  1.19 (0.47, 3.05) |
| Marital status | Partnered  Single | Ref  1.14 (0.69, 1.87) | Ref  1.16 (0.56, 2.38) | Ref  1.21 (0.61, 2.39) |
| History of fracture | Yes | **2.13 (1.16, 3.91)** | 1.86 (0.66, 5.20) | 1.17 (0.36, 3.78) |
| Arthritis | Yes | 1.23 (0.30, 5.05) | 1.66 (0.23, 12.17) | Not enough events |
| Menopausal status | Yes | 0.86 (0.45, 1.65) | 1.20 (0.47, 3.04) | 1.03 (0.41, 2.59) |
| Other condition | Yes | 1.49 (0.47, 4.79) | 1.32 (0.18, 9.71) | 1.15 (0.16, 8.42) |
| Bone medications prior year* | Yes | 0.97 (0.53, 1.77) | 1.63 (0.73, 3.64) | 0.74 (0.27, 2.06) |
| Steroids prior year | Yes | 1.12 (0.67, 1.88) | 1.48 (0.69, 3.17) | 0.90 (0.38, 2.11) |
| Antiepileptics prior year | Yes | 0.65 (0.30, 1.40) | 0.76 (0.23, 2.45) | 0.65 (0.20, 2.10) |
| Antidepressants prior year | Yes | 1.15 (0.80, 1.64) | **2.01 (1.14, 3.56)** | 1.64 (0.96, 2.79) |
| Anxiolytics/sedatives/hypnotics prior year | Yes | **1.77 (1.09, 2.87)** | **3.21 (1.66, 6.20)** | 1.62 (0.76, 3.45) |
| Diabetes | Yes | 1.00 (0.68, 1.46) | 0.98 (0.53, 1.83) | 1.16 (0.66, 2.05) |
| Kg weight loss prior year | Per 1kg decrease | 0.96 (0.87, 1.06) | 0.99 (0.83, 1.18) | 0.99 (0.83, 1.17) |

**Supplement 4:** Receiver operating characteristic curves for (a) any and (b) major fracture

b)

a)


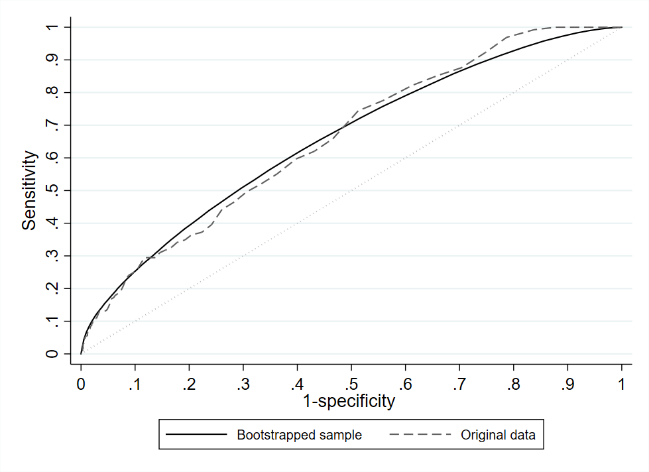

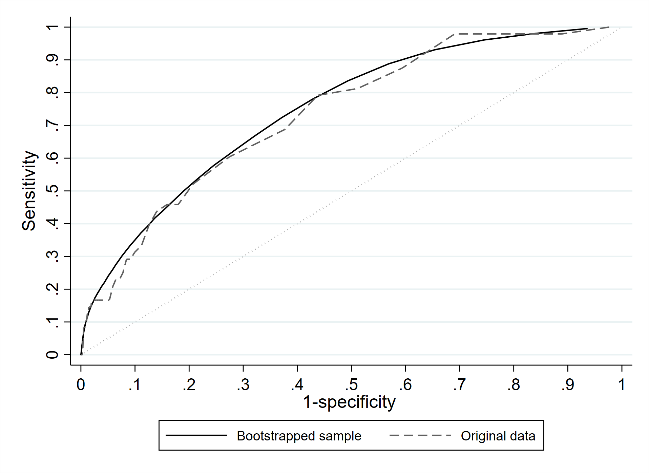

Supplement: Supplementary file 1 — Appendix S1. Supporting Information [file JBMR-36-2153-s001.docx]
